# Glucosinolates, Ca, Se Contents, and Bioaccessibility in *Brassica rapa* Vegetables Obtained by Organic and Conventional Cropping Systems

**DOI:** 10.3390/foods11030350

**Published:** 2022-01-26

**Authors:** Fernando Cámara-Martos, Sara Obregón-Cano, Antonio de Haro-Bailón

**Affiliations:** 1 Departamento de Bromatología y Tecnología de los Alimentos, Universidad de Córdoba, Campus Universitario de Rabanales, Edificio C-1, 14014 Cordoba, Spain; 2 Departamento de Mejora Genética Vegetal, Instituto de Agricultura Sostenible (IA-CSIC), 14004 Cordoba, Spain; saraobregon@ias.csic.es (S.O.-C.); adeharobailon@ias.csic.es (A.d.H.-B.)

**Keywords:** organic farming, conventional farming, trace elements, glucosinolates, DRIs

## Abstract

In northwest Spain and Portugal, there is a long tradition of cultivating *B. rapa* subsp. *rapa* to obtain turnip greens and turnip tops. *Brassica rapa* L. subsp. *rapa* (turnip greens and turnip tops) were grown under conventional and organic conditions in two Farms in southern Spain. Glucosinolatescontents were higher in Brassicas grown under conventional conditions than those grown under organic ones. Average Ca total and bioaccessible contents ranged between 14.6–23.4 mg/g; 8.9–12.0 mg/g for turnip greens and 6.4–8.9 mg/g; 4.3–4.8 mg/g for turnip tops. According to these concentrations, an intake of 100–200 g (fresh weight) of the studied *Brassica rapa* fulfills Ca dietary reference intakes (DRI) (considering the total content data) and complies with 72–100% Ca DRI percentage (considering the bioaccessible data). Se concentrations ranged between 0.061–0.073 µg/g and 0.039–0.053 µg/g for turnip greens and turnip tops respectively. Se bioaccessibility values were high, with percentages of around 90%. Finally, the total glucosinolate content ranged between 13.23–21.28 µmol/g for turnip greens and 13.36–20.20 µmol/g for turnip tops. In general, the bioaccessibility of the total glucosinolates analyzed in this study was high, with mean values of around 73% and 66% for turnip greens and turnip tops, respectively. *Brassica rapa* vegetables grown under both organic and conventional conditions in southern Spain are an excellent dietary source of Ca, Se, and glucosinolates with a high bioaccessibility.

## 1. Introduction

Meat and dairy products in the food industry represent a significant portion of anthropogenic greenhouse gas emission [1]. As a consequence of this, and of following healthier dietary patterns, a larger number of consumers are being encouraged to consume more vegetables [2]. Plant species belonging to the *Brassicaceae* family were one of the first plant groups cultivated and domesticated by humanity. Within this family, we found relevant foods of vegetable origin such as broccoli, cabbage, cauliflower, mustard, rapeseed, rocket, and turnip, and it is one of the most economically important plant families in the world [3,4]. Those vegetables are also an excellent source of inorganic micronutrients with a high bioavailability and of health-promoting phytochemicals, such as glucosinolates [5,6,7].

Thus, glucosinolates are some of the most important secondary metabolites in the *Brassicaceae* family. These sulfur compounds are hydrolyzed by the myrosinase enzyme (present endogenously in these plants) producing hydrolysis breakdown products (isothiocyanates), which have a protective and preventive effect against several kinds of cancer [6,8,9,10]. Among the inorganic micronutrients, Se plays a relevant role in human nutrition, forming part of the active site of glutathione peroxidase, one of the main antioxidant enzymes [11,12]. This trace element can interact with S (of glucosinolates) due to their similar physical and chemical characteristics and their close association with plant metabolism [13]. Secondly, these plants have proven to be a good dietary source of Ca [14].

*Brassica rapa L.* is an economically important species belonging to the *Brassicaceae* family. This species, which grows naturally from the western Mediterranean region to Central Asia, can be used as oil, and the roots, leaves, stems, and flowers are consumed as vegetables in many parts of the world [15,16]. In northwest Spain and Portugal, there is a long tradition of cultivating *B. rapa* subsp. *rapa* to obtain turnip greens and turnip tops. Turnip greens are the young leaves harvested in the vegetative growth period, and turnip tops are the fructiferous stems with flower buds and the surrounding leaves that are consumed while still green. However, their cultivation has been limited in southern areas or in the Mediterranean basin, probably due to a lack of adaptation [7]. Nevertheless, this type of crop could have a place within the Mediterranean diet, based mainly on a greater consumption of fruit and vegetables. With this objective in mind, a breeding program in *B. rapa* subsp. *rapa* has been developed in the south of Spain to obtain varieties adapted to the environmental conditions of this area but preserving similar nutritional properties to those produced in their original region [7,17].

These turnip greens and turnip tops can also be grown under organic conditions [18]. This cultivation system is experiencing considerable growth, with an increase of around 250% in the past 10 years [19]. In Spain, the area under organic cultivation has increased in the last 10 years from 10,156 to 22,482 hectares. At the same time, the production of organic vegetables in Spain has also experienced a considerable increase in the last 5 years, from 220,983 to 567,599 tons [20]. The consumption of these Brassicas produced locally has an additional environmental benefit due to their lower transport costs.

Some authors have pointed out that organic foods contain higher concentrations of nutritionally beneficial trace elements and phytochemicals such as glucosinolates [21,22]. However, the data existing in the bibliography on this topic are inconclusive [23], and it is difficult to make a valid comparison between both vegetable groups due to the limited availability of well-controlled or paired studies [24].

In addition, bioaccessibility is another variable that must be considered when evaluating the nutritional value of a food [25]. It is considered as the fraction of micronutrient or bioactive compound initially present which is solubilized in the intestinal lumen and consequently, it would be susceptible to be taken up by enterocites. Although an attempt has been made to achieve a certain standardization in the methodology to assess bioaccessibility [26,27], there is no universally accepted consensus. However, all of them reproduce the physiological conditions that occur in the stomach and small intestine during the human digestive process.

Considering all the above, the objectives of this research were to compare the total contents and bioaccessibility of Ca, Se, and glucosinolates in turnip greens and turnip tops (*Brassica rapa*) grown under both conventional and organic conditions on two different experimental farms located in southern Spain. This information will promote a better understanding of the beneficial roles of these vegetables within the framework of a healthier diet.

## 2. Materials and Methods

### 2.1. Plant Material

One cultivar (Br AR-02) from *Brassica rapa* L. subsp. *rapa* was chosen based on previous studies showing its good adaptation to the Mediterranean environment. This cultivar was obtained by us at the Institute for Sustainable Agriculture (IAS-CSIC) after several cycles of breeding for agronomic performance and high content in beneficial glucosinolates.

**Figure 1:**
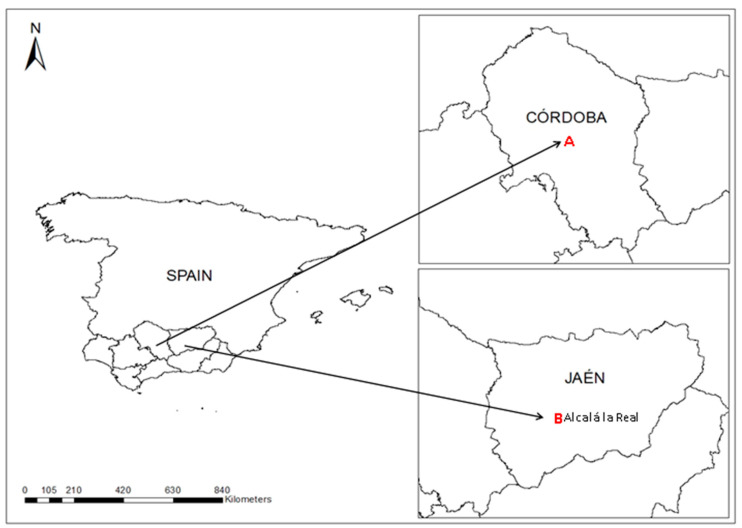
Location of two experimental farms in the southern Spain.

This material was sown and cultivated during the season 2019–2020 in two farms (I and II) in southern Spain (see Figure 1).

Farm I (37°51′ N, 4°48′ W) is located in Córdoba, bordering the Guadalquivir River, in a first terrace position (altitude of 106 m), with a deep soil (Typic Xerofluvent) of sandy-loam texture with a high pH (around 8), intermediate organic matter content (1.6%), and high carbonate content (17%). The experimental plot size for conventional cultivation of *Brassica rapa* plants on Farm I was 25 m × 25 m. The climate is typically continental Mediterranean (Csa in Köppen’s climate classification), with relatively cold winters, intensely hot dry summers, and mean annual precipitations of 650 mm. On Farm I, the *Brassica rapa* cultivar was only grown under conventional conditions with herbicides and mineral fertilization being applied pre-sowing. An herbicide with triflularin as its active matter was used at a dose of 1.5 L/ha. Moreover, before sowing, a basic dressing with 8-15-15 bottom fertilizer was added at a rate of 600 kg/ha. A top dressing (cover fertilization) with 300 kg/ha of ammonium nitrate was applied at the end of the winter, with the resumption of vegetative growth.

Farm II is located in the municipal district of Alcalá la Real (Jaén) (37°27′ N 3°55′ W, Spain) in the Sub-Baetic zone, next to the Velillos River (altitude 920 m) with a moderately stony structure and clay loam texture (Xerofluvent-Fluvisol calcareous) with a high pH (8.2), high organic matter content (3%), and a high carbonate content (16%). The experimental plot size for the conventional and ecological cultivation of *Brassica rapa* cultivar on Farm II was 25 m × 25 m each. Both experimental plots were close together and separated only by a 2 m-wide border. The climate is typically continental Mediterranean (Csa in Köppen’s climate classification), with short, very hot, arid, and mostly cloudless summers, and very cold, partially cloudy winters. The mean annual precipitations were 650 mm on this farm; the *Brassica rapa* cultivar was grown both under conventional and organic conditions.

The conventional cultivation conditions on Farm II were similar to those of Farm I. In organic cultivation, neither herbicides nor mineral fertilizers were added. Instead, only treatment with a mixture of goat and sheep manure was applied at a rate of 3 kg/m^2^.

When the young leaves (turnip greens) or fructiferous stems with flower buds (turnip tops) reached their optimum moment of consumption (from 3 to 5 months after sowing, respectively), five turnip green samples, and, two months later, five turnip top samples from ten individual plants were harvested, pooled, and processed for chemical analysis. Turnip green samples (*n* = 50) and turnip top samples (*n* = 50) were thoroughly washed with tap water to eliminate dirt, and they were finally rinsed with deionized water. Then, they were frozen and stored at −80 °C until freeze-drying, which was done in Telstar^®^ model Cryodos-50 equipment (Telstar, Terrasa, Spain). The freeze-dried samples were ground in a Janke and Kunkel Model A10 mill (IKA-Labortechnik, Staufen, Germany) for about 20 s, and stored in a desiccator until their analysis.

### 2.2. Material and Reagents

All the reagents were analytical-reagent grade. Ultrapure water (18 MΩ/SCF), prepared with a Milli-Q Reference Water Purification (Millipore, Madrid, Spain), was used throughout. All glassware and plastic containers were soaked in 50% nitric acid overnight and 20% hydrochloric acid, also overnight, and rinsed three times with de-ionized water prior to use. Sodium bicarbonate (97%) was obtained from Scharlau (Barcelona, Spain), magnesium nitrate hexahydrate (98%), and magnesium oxide (98%) from Alfa Aesar (Kandel, Germany). Hydrochloric acid (35%) and hyperpure nitric acid (65%) were obtained from Panreac (Barcelona, Spain). Lanthanum chloride was supplied by Perkin Elmer (Madrid, Spain).

Digestive enzymes and bile salts were supplied by Sigma-Aldrich Co. (St. Louis, MO, USA). Working solutions of these enzymes were prepared immediately before use. Pepsin (3.2 g, P-7000 from porcine gastric mucosa) was dissolved in 20 mL of HCl (0.1 M). Pancreatin (0.6 g, P-3292 from porcine pancreas) and bile salts (3.9 g, B-8756 of porcine origin) were dissolved in 150 mL NaHCO_3_ (0.1 M).

### 2.3. Procedure for In Vitro Gastrointestinal Digestion (Solubility Assay)

The procedure described by Cámara et al. [25], with slight modifications, was used to estimate Ca, Se, and glucosinolates bioaccessibility (solubility). The first stage of the assay imitates the gastric phase. Thus, 3 g of each freeze-dried *Brassica rapa* sample (turnip green or turnip top) was homogenized with 22 mL of deionized water, and the pH was adjusted to 2 with 6 M HCl. Then, 0.5 g of pepsin solution per 100 g of homogenized (sample and deionized water mixture) was added (corresponding to 0.125 g of porcine pepsin per 3 g of freeze-dried sample). The mixture was then incubated for 2 h at 37 °C in a shaking water bath (HSB-2000 Shaking Bath; E-Chrom Tech CO., LTD, Taipei, Taiwan). 

For the intestinal stage, the pH was adjusted to 5 by adding 1 M NaHCO3. Then, 6.3 mL of a mixture of pancreatin and bile salts (corresponding to 0.025 g of pancreatin and 0.160 g of bile salts per 3 g of freeze-dried sample) was added to each test tube, that was incubated for a further 2 h.

Finally, the pH was adjusted to 7.2 with 0.5 M NaOH. Aliquots of the digested sample were transferred to polypropylene centrifuge tubes (50 mL, Costar Corning Europe, Badhoevedorp, The Netherlands) that were then centrifuged for 1 h at 4000 rpm and 3 °C. (Eppendorf Centrifuge 5810 R). The supernatant (soluble fraction) was collected to determine the concentration of trace elements and glucosinolates as specified in the following section.

### 2.4. Ca and Se Determination

To determine the total Ca and Se of *Brassica rapa* samples, a well-established and validated protocol from previous studies was followed [12,14,18]. Thus, 0.5 g of freeze-dried turnip greens or turnip tops were weighed in a porcelain crucible. To prevent Se volatilization, samples were treated with 5 mL of 7 M HNO_3_ and 1.5 mL of ashing aid suspension (20% *w*/*v* MgNO_3_ and 2% *w*/*v* MgO). The mixture was evaporated on a hot plate at 80 °C until total dryness. Subsequently, samples were incinerated in a muffle furnace at 460 °C for 16 h. The ashes obtained were bleached after cooling by adding 200 µL of hyperpure HNO_3_ and 2 mL of deionized water, heating to dryness, and placing in a muffle furnace for 1 h more. Ashes were recovered with 100 µL of hyperpure HNO_3_, made up to 10 mL with deionized water. 

Ca (λ = 422.7 nm; slit width = 0.7 nm) was determined by flame absorption atomic spectroscopy (FAAS) with a Varian Spectra AA-50 B model, equipped with single element hollow cathode lamps and a standard air-acetylene flame. LaCl_3_ was added to the mineral solution at a final concentration of 2%, to avoid interference by phosphate.

Se was analyzed by atomic fluorescence spectroscopy (Millennium Excalibur Instrument, PSA Analytical). This equipment is equipped with a Se discharge hollow-cathode lamp (λ = 196.0 nm; current = 20 mA) (Photron) and a hygroscopic membrane drying tube (Permapure). Online hydride generation was performed by adding NaBH_4_ 0.7% *w/v* (in NaOH 0.1 M) and HCl 4.5 M solutions by means of a peristaltic pump at a flow rate of 10 mL/min. Argon was used as a carrier gas (300 mL/min) to transport the Se hydrides to the gas–liquid separator.

Standard solutions for measuring Ca and Se were prepared immediately before use by dilution with distilled deionized water of 1000 mg/L standard solutions (Certipur-Merck, Darmstadt, Germany). The accuracy and precision of the method used in determining Ca and Se concentrations were validated by recovery experiments using certified reference materials (see Table 1).

### 2.5. Glucosinolates Analysis

The glucosinolates (total and bioaccessible) were analyzed by high-performance liquid chromatography (HPLC). Extraction and desulphation of glucosinolates from the freeze-dried samples were performed according to the method developed by Font et al. [28]. About 100 mg dry weight of the lyophilized sample was precisely weighed, and a two-step glucosinolate extraction was carried out in a water bath at 75 °C to inactivate myrosinase. In the first step, the sample was heated for 15 min in 2.5 mL 70% aqueous methanol and 200 µL 10 mM glucotropaeolin (benzyl glucosinolate) from PhytoPlan^®^ (Heidelberg, Germany, 3403.99) was added as internal standard. A second extraction was applied after centrifugation (5 min, 5 × 10^−3^ g) by using 2 mL of 70% aqueous 126 methanol (CAS: 67-56-1). One milliliter of the combined glucosinolate extracts was pipetted onto the top of an ion-exchange column containing 1 mL Sephadex DEAE-A25 (Sigma-Aldrich, St. Louis, MO, USA, A25120). Desulphation was carried out by the addition of 75 µL of purified sulphatase (E.C. 232-772-1, type H-1 from *Helix pomatia*, Sigma-Aldrich, St. Louis, MO, USA, S9751) solution. Desulphated glucosinolates were eluted with 2.5 mL (0.5 mL × 5) Milli-Q (Millipore) ultra-pure water and analyzed with a Model 133 600 HPLC instrument (Waters) equipped with a Model 486 UV tunable absorbance detector (Waters) at a wavelength of 229 nm. Separation was carried out by using a 135 Lichrospher 100 RP-18 in Lichrocart 125−4 column, 5 µm particle size (Merck). HPLC 136 solvents and gradient were fixed according to the ISO protocol (ISO 9167-1). The mobile phase was a mixture of (A) acetonitrile (HPLC grade) and (B) acetonitrile/water (20:80). The flow rate was 1 mL/min in a linear gradient starting with 99% solvent A + 1% solvent B for 1 min, reaching 1% A + 99% B at 20–23 min, and return to 99% A + 1% B at 28 min, and remaining at 99% + 1% B during 10 min. The HPLC chromatogram was compared to the desulpho-glucosinolate profile of three certified reference materials recommended by UE and ISO (CRMs 366, 190 and 367), to compare the peaks with the corresponding glucosinolate. Data were corrected for UV response factors for the different types of glucosinolates (ISO 9167−1).

The amount of each individual glucosinolate present in the sample was calculated with the standard internal method as recommended by the ISO protocol and expressed as µmol/g of dry weight. The total glucosinolate content was calculated as the sum of all the individual glucosinolates present in the sample.

To analyze the glucosinolate content in the soluble fraction, the procedure described by Camara et al. [14] was followed.

## 3. Results and Discussion

### 3.1. Calcium

Average Ca contents ranged between 14.6–23.4 mg/g for turnip greens and 6.4–8.9 mg/g for turnip tops. The Ca content of the samples of turnip greens grown on Farm I was significantly higher than that of the samples of turnip greens grown on Farm II. No significant differences were found between samples of turnip greens grown under organic and conventional conditions on Farm II (Table 2). On the other hand, the average Ca content of the turnip top samples was lower than the average Ca content of the turnip greens samples and no significant differences were detected between the Ca content of the turnip top samples in the three groups (conventional Farm I, conventional Farm II, organic Farm II) (Table 3). A similar result was obtained in a previous study [18] for other micronutrients (Co, Cr, Cu, Fe, Mn, and Zn) in *Brassicaceae* species, showing that, together with farming systems, the trace element concentrations in foodstuffs depend on many other factors, including soil characteristics, seasonal influences, genetic factors, interactions between the elements and pollution from anthropogenic sources.

The high Ca concentrations reported in the present study are in agreement with those provided in previous works: 19.7 mg/g [29]; 4.3–18.5 mg/g [30]; 6.2–19.5 mg/g [14]. In addition, it has been pointed out that Brassicas are a good dietary source of bioaccessible Ca due to their low content of some chelating agents for Ca such as oxalates [31]. Indeed, previous studies [32] reported much lower oxalate concentrations (50–95 mg/100 g) in Brassica species (such as brussels sprouts, broccoli, green and white cauliflower) than those reported for other green leafy vegetables such as Swiss chard (747–816 mg/100 g) [33] or spinach (1634–2285 mg/100 g) [34].

In the present study, the average concentrations of bioaccessible Ca ranged between 8.9–12.0 mg/g for turnip greens and 4.3–4.8 mg/g for turnip tops (Table 3 and Table 4). These results are slightly higher than those reported in a previous study for Brassicas (3.1–7.2 mg/g) [14]. In that study, the authors also proved that Brassicas presented a similar bioaccessibility to skimmed milk powder and was an alternative source of Ca for people with dairy product intolerance. According to our data, an intake of 100–200 g (fresh weight) of the *Brassica rapa* studied fulfills Ca DRI (900 mg/day) [35] (considering the total content data) and complies with 72–100% Ca DRI (considering the bioaccessible data). All of this reinforces the idea that Brassicas are an excellent dietary source of highly available Ca.

### 3.2. Selenium

*Brassicaceae* species can accumulate high concentrations of Se with little or no ostensible impairment to the plant [36]. In this study, Se concentrations ranged between 0.061–0.073 µg/g for turnip greens and 0.038–0.043 µg/g for turnip tops. There were no statistically significant differences for Se content between turnip green samples of plants grown under conventional or organic conditions (Table 4), and the same occurred for turnip tops (Table 5). In addition, these contents are in agreement with those reported in a previous study (0.053 ± 0.018 µg/g) for *Brassica rapa* [14].

Due to chemical similarities between Se and S (the two elements are part of the same group of the periodic table), it has been highlighted that an increase in Se accumulation in plants might affect the synthesis of S-related compounds [13], among them glucosinolates. Charron et al. [37] and Toler et al. [38], in *Brassica oleracea* species, observed a decrease in total glucosinolate concentrations when different hydroponic solutions were supplemented with increasing concentrations of sodium selenate. Conversely, other authors found that fertilization with Se did not produce any change in glucosinolate concentrations of *Brassica oleracea* species [39,40].

Average bioaccessible Se concentrations ranged between 0.059–0.067 µg/g for turnip greens (Table 5) and between 0.038–0.047 µg/g for turnip tops (Table 4). Bioaccessibility values in the analyzed Brassicas were high, with percentages of around 90%, showing these vegetables (in the same way as Ca) to be a good dietary source of bioaccessible inorganic micronutrients.

### 3.3. Glucosinolates

Five glucosinolates (Supplementary file) were identified and quantified: three aliphatic-methionine-derived compounds (progoitrin, gluconapin, and glucobrassicanapin), and two indolic tryptophan-derived compounds (glucobrassicin and 4-methoxyglucobrassicin) (Table 6 and Table 7). Turnip greens and turnips top grown under organic and conventional cropping systems have a similar glucosinolate profile but differ in their concentrations. Aliphatic glucosinolates were predominant in all the samples, representing more than 90% of total glucosinolates content, and especially gluconapin, that prevailed in all the turnip greens (from 66.87 to 76.84% of the total glucosinolate content), and turnip tops (from 79.31 to 79.57% of the total glucosinolate content).

The total glucosinolate content of turnip greens ranged between 13.23–1.28 μmol/g dry weight, and was significantly higher (*p* < 0.05) in turnip greens grown on Farm I than that of turnip greens grown on Farm II (Table 6). No significant differences for total glucosinolate content were found between the samples of turnip greens grown under either organic or conventional conditions on Farm II. The content of the predominant glucosinolate, gluconapin, was also higher in turnip greens grown under the conventional systems on Farms I and II than that of turnip greens grown under organic conditions (Farm II). No significant differences were observed for progoitrin content in turnip greens under either conventional or organic cultivation conditions.

These results are comparable to those of previous works on glucosinolate content for this species grown under conventional cropping system: Padilla et al. [41] studied a germplasm collection of 113 entries of turnip greens and found a total glucosinolate content ranging from 11.80 to 74.00 µmol/g dry weight. Cámara-Martos, et al. [14] reported a total glucosinolate content value of 11.20 µmol/g dry weight in leaf samples of *B. rapa*, and Soengas et al. [42] reported high glucosinolate content values in tops and leaves from five accessions of this species (48.90 and 40.92 µmol/g dw, respectively).

The total glucosinolates content of turnip tops ranged between 13.36–20.20 µmol/g dry weight, and it was significantly higher (*p* < 0.05) in turnip tops grown under conventional systems than that of turnip tops grown under organic ones (Table 7). There were significant differences (*p* < 0.05) in the glucosinolate content of the three groups of samples, the highest being in turnip tops grown under conventional system on Farm II, followed by turnip tops grown on Farm I, and the lowest content of glucosinolate was found in turnip tops grown under organic cultivation conditions. This fact could be an important key to selecting cropping systems, because of the beneficial properties of glucosinolates for human health and consumption [6,10]. Progoitrin content in turnip tops grown under conventional conditions was also significantly higher (*p* < 0.05) than that of turnip tops grown under organic cultivation systems.

For the remaining glucosinolates found (glucobrassicanapin, glucobrassicin, and 4-methoxiglucobrassicin), there were no significant differences or any clear trend between turnip tops from plants grown in the two cultivation systems.

Some authors have shown a higher concentration of glucosinolates in Brassica species grown under organic conditions than in conventional systems [22]. It seems that in organic Brassicas the concentration of bioactive compounds such as vitamin C, phenols, and glucosinolates increased as a consequence of a defense mechanism to counteract the oxidative damage triggered by stress conditions under organic agricultural practices [43]. Conversely, Conversa et al. [44] found lower glucosinolate levels compared with the conventionally grown product in two early-flowering landraces of cima di rapa “Cimagranda” and “Riccia di San Manzano” (*Brassica rapa* L. subsp. *sylvestris*).

These authors justify this result as being due to a lower N:S ratio in the conventional crop compared to the organic one. Thus, the application of mineral fertilizer to the conventional crop decreases the availability of soil nitrogen, while increasing that of sulfur due to the application of ammonium sulfate fertilizer to the conventional crop [44]. The result was this increase in the concentration of total glucosinolates in Brassicas, which is in agreement with the results reported in the present study.

In relation to bioaccessibility, both turnip greens and turnip tops showed the same pattern for the major glucosinolates, with the bioaccessibility of total glucosinolates, gluconapine and progoitrin being significantly higher in plants grown under conventional conditions than in plants grown under organic ones (Table 8 and Table 9). For the remaining glucosinolates analyzed (glucobrassicanapin, glucobrassicin, and 4-methoxyglucobrassicin), no clear tendencies or no significant differences in bioaccessibility were found between the plants grown in the two cultivation systems (Table 8 and Table 9).

In general, the bioaccessibility of the total glucosinolates analyzed in this study was high, with mean values of around 73% and 66% for turnip greens and turnip tops, respectively. This implies that most of the glucosinolates initially present in the leaves of *Brassica rapa* varieties would be capable of reaching human enterocytes, resisting the degradation processes of digestive enzymes, including its own myrosinase enzyme. These bioaccessibility values were higher than those reported in a previous study [14] for Brassica rapa (44%), probably due to an equally higher initial glucosinolate concentration in the present study.

Bioaccessibilities of predominant glucosinolates (gluconapin, progoitrin, and glucobrassicanapin) were also high, with medium values of 78, 72, and 67%, respectively, in turnip greens, and 67, 49, and 78%, respectively, in turnip tops. All these glucosinolates are categorized as being aliphatic ones. From a nutritional point of view, this was an important finding because data mining suggested that, of the different dietary-derived glucosinolate subgroups, aliphatic glucosinolates showed the strongest inverse association with cancer risk.

Navarro et al. [45] indicated that supplementing with cruciferous vegetables (single dose-7 g cruciferous/kg body weight or twice-dose-14 g cruciferous/kg body weight), a basal diet devoid of fruit and vegetables, lowered bilirubin concentrations dose dependently after 14 days. Serum bilirubin concentrations were measured to monitor UDP-glucuronosyl-transferase activity. These enzymes catalyze the transfer of glucuronyl groups to endogenous and exogenous molecules (drug and dietary carcinogens) to produce more polar molecules which reduce their toxicity, as they were more easily excreted [46]. However, in that study, neither the type of cruciferous nor the concentration of glucosinolates present in them were indicated.

Furthermore, Traka et al. [47] found considerable evidence for the perturbation of several signaling pathways associated with carcinogenesis and inflammation after 12 months’ consumption of 400 g weekly of steamed broccoli (with 10.6 µmol/g dry weight glucoraphanin and 3.6 µmol/g dry weight glucoiberin, precursors of the isothiocyanates sulforaphane, and iberin, respectively). According to these authors, broccoli intervention was associated with the perturbation of TGFβ1, EGF, and insulin signaling, each of which has been associated with prostate carcinogenesis [48,49,50], in addition to carcinogenesis at other sites [51,52]. In our study, the main glucosinolates found in *Brassica rapa* plant species were different, i.e., gluconapin, glucobrassicanapin, and progoitrin, and consequently, the allyl isothiocyanates derived from their hydrolysis. Nevertheless, the gluconapin (9.65–16.02 µmol/g dw), glucobrassicanapin (0.70–4.22 µmol/g dw), and progoitrin (0.38–2.26 µmol/g dw) concentrations analyzed are quite similar to those reported by Traka et al. [47], which seems to infer that two weekly rations of 150–200 g of turnip tops or turnip greens could exert a similarly beneficial effect.

It has also been pointed out that myrosinase plays a key role in the metabolism and bioaccessibility of glucosinolates [53,54]. As a result, the beneficial effect of glucosinolates ingested from natural vegetable sources is not the same as when they are provided as diet supplements. Thus, Clarke et al. [54] studied the effect of the consumption of 40 g of broccoli sprouts (150 µmol glucoraphanin and 71 µmol glucoerucin) in 16 healthy subjects (19–50 years). The amount of metabolites (sulforaphane and erucin) coming from glucosinolate hydrolysis per myrosinase enzyme was monitored in the plasma and urine of the volunteers. It was found that the sulforaphane and erucin concentrations were higher when glucosinolates were ingested through broccoli sprouts than if they were consumed in the same dose as dietary supplements (pills). All this indicates that the best way to provide the human body with these compounds is through the natural sources (i.e., vegetables) in which they are found.

## 4. Conclusions

Our results show that the fact that *Brassica rapa* vegetables are grown under organic conditions does not guarantee a higher content in beneficial compounds for human health such as Ca, Se, and glucosinolates. On the contrary, these compounds tend to be more abundant in the conventional system. In any case, turnip greens and turnip tops cultivated under both organic and conventional conditions in southern Spain have proven to be an excellent dietary source of Ca, Se, and glucosinolates with a high bioaccessibility. Therefore, the incorporation of turnip greens and turnip tops into the Mediterranean cuisine and their consumption should be encouraged within the framework of a more sustainable and healthier diet.

## Figures and Tables

**Table 1:** Analysis of certified references materials (mean ± standard deviation).

Element	Mussel Tissue ERM-C278k			White Cabbage BCR-679		
(mg/Kg)	Certified	Found	Recovery (%)	Certified	Found	Recovery (%)
Ca	-	-	-	7768 ± 655 *	7624 ± 293	98
Se	1.62 ± 0.12	1.50 ± 0.07	97	-	-	-

* Indicative value.

**Table 2:** Ca total and bioaccessible (mg/g dry weight) in turnip greens (mean ± standard deviation).

Sample	Total Ca (mg/g)	Bioaccessible Ca (mg/g)
Conventional turnip greens (Farm I)	23.44 ± 0.58 ^b^	12.02 ± 0.50 ^c^
Conventional turnip greens (Farm II)	14.57 ± 0.96 ^a^	10.10 ± 0.44 ^b^
Organic turnip greens (Farm II)	15.99 ± 2.95 ^a^	8.88 ± 0.25 ^a^

Within each column means with different lowercase letters (a–c) are significantly different at *p* < 0.05 according to the analysis of variance (ANOVA) and Duncan test.

**Table 3:** Ca total and bioaccessible (mg/g dry weight) in turnip tops (mean ± standard deviation).

Sample	Total Ca (mg/g)	Bioaccessible Ca (mg/g)
Conventional turnip tops (Farm I)	8.20 ± 0.04 ^a^	4.82 ± 0.83 ^a^
Conventional turnip tops (Farm II)	6.44 ± 2.14 ^a^	4.27 ± 0.40 ^a^
Organic turnip tops (Farm II)	8.95 ± 2.37 ^a^	4.82 ± 0.17 ^a^

Within each column means with the same lowercase letter (a) are not significantly different at *p* < 0.05 according to the analysis of variance (ANOVA) and Duncan test.

**Table 4:** Se total and bioaccessible (µg/g dry weight) in turnip greens (mean ± standard deviation).

Sample	Total Se (µg/g)	Bioaccessible Se (µg/g)
Conventional turnip greens (Farm I)	0.061 ± 0.011 ^a^	0.067 ± 0.011 ^a^
Conventional turnip greens (Farm II)	0.073 ± 0.001 ^a^	0.059 ± 0.002 ^a^
Organic turnip greens (Farm II)	0.064 ± 0.020 ^a^	0.059 ± 0.002 ^a^

Within each column means with the same lowercase letter (a) are not significantly different at *p* < 0.05 according to the analysis of variance (ANOVA) and Duncan test.

**Table 5:** Se total and bioaccessible (µg/g dry weight) in turnip tops (mean ± standard deviation).

Sample	Total Se (µg/g)	Bioaccessible Se (µg/g)
Conventional turnip tops (Farm I)	0.039 ± 0.003 ^a^	0.038 ± 0.001 ^a^
Conventional turnip tops (Farm II)	0.038 ± 0.005 ^a^	0.040 ± 0.002 ^b^
Organic turnip tops (Farm II)	0.043 ± 0.003 ^a^	0.047 ± 0.002 ^c^

Within each column means with different lowercase letters (a–c) are significantly different at *p* < 0.05 according to the analysis of variance (ANOVA) and Duncan test.

**Table 6:** Content (µmol/g dry weight) of total and individual glucosinolates in turnip greens (mean ± standard deviation).

Sample	Total	PRO	GNA	GBN	GBS	4OMGBS	Others
Conventional turnip greens (Farm I)	21.28 ± 1.90 ^b^	2.09 ± 0.97	14.23 ± 0.52 ^b^	4.22 ± 0.20 ^c^	0.25 ± 0.11 ^a^	0.22 ± 0.02 ^a^	0.27 ± 0.08 ^a^
Conventional turnip greens (Farm II)	16.54 ± 2.06 ^a^	1.27 ± 0.49	12.71 ± 0.84 ^b^	0.82 ± 0.06 ^a^	0.37 ± 0.16 ^a^	0.50 ± 0.51 ^a^	0.87 ± 0.23 ^b^
Organic turnip greens (Farm II)	13.23 ± 0.89 ^a^	1.01 ± 0.54	9.65 ± 1.00 ^a^	1.90 ± 0.24 ^b^	0.19 ± 0.08 ^a^	0.23 ± 0.01 ^a^	0.25 ± 0.10 ^b^

PRO (Progoitrin), GNA (Gluconapin), GBN (Glucobrassicanapin), GBS (Glucobrassicin), 4OMGBS (4-Methoxyglucobrassicin). Within each column means with different lowercase letters (a–c) are significantly different at *p* < 0.05 according to the analysis of variance (ANOVA) and Duncan test.

**Table 7:** Content (µmol/g dry weight) of total and individual glucosinolates in turnip tops (mean ± standard deviation).

Sample	Total	PRO	GNA	GBN	GBS	4OMGBS	Others
Conventional turnip tops (Farm I)	17.86 ± 1.63 ^b^	2.26 ± 0.77 ^b^	14.18 ± 0.81 ^b^	0.70 ± 0.06 ^a^	0.19 ± 0.01 ^a^	0.15 ± 0.01 ^a^	0.38 ± 0.04 ^b^
Conventional turnip tops (Farm II)	20.20 ± 1.59 ^b^	1.84 ± 0.56 ^b^	16.02 ± 1.01 ^c^	1.72 ± 0.15 ^c^	0.17 ± 0.07 ^a^	0.29 ± 0.13 ^a^	0.16 ± 0.02 ^a^
Organic turnip tops (Farm II)	13.36 ± 0.04 ^a^	0.38 ± 0.12 ^a^	10.63 ± 0.23 ^a^	1.40 ± 0.07 ^b^	0.26 ± 0.23 ^a^	0.34 ± 0.02 ^a^	0.34 ± 0.02 ^b^

PRO (Progoitrin), GNA (Gluconapin), GBN (Glucobrassicanapin), GBS (Glucobrassicin), 4OMGBS (4-Methoxyglucobrassicin). Within each column means with different lowercase letters (a–c) are significantly different at *p* < 0.05 according to the analysis of variance (ANOVA) and Duncan test.

**Table 8:** Bioaccessible content (µmol/g dry weight) of total and individual glucosinolates in turnip greens (mean ± standard deviation).

Sample	Total	PRO	GNA	GBN	GBS	4OMGBS	Others
Conventional turnip greens (Farm I)	15.4 ± 0.4 ^c^	1.20 ± 0.10 ^b^	10.7 ± 0.3 ^b^	3.11 ± 0.05 ^c^	0.20 ± 0.01 ^b^	0.10 ± 0.03 ^a^	0.10 ± 0.01 ^a^
Conventional turnip greens (Farm II)	12.2 ± 0.1 ^b^	0.76 ± 0.05 ^a^	10.5 ± 0.1 ^b^	0.74 ± 0.03 ^a^	0.09 ± 0.00 ^a^	0.10 ± 0.03 ^a^	0.10 ± 0.03 ^a^
Organic turnip greens (Farm II)	9.5 ± 0.3 ^a^	0.85 ± 0.05 ^a^	7.3 ± 0.3 ^a^	0.99 ± 0.03 ^b^	0.10 ± 0.00 ^a^	0.10 ± 0.02 ^a^	0.09 ± 0.01 ^a^

PRO (Progoitrin), GNA (Gluconapin), GBN (Glucobrassicanapin), GBS (Glucobrassicin), 4OMGBS (4 Methoxyglucobrassicin). Within each column means with different lowercase letters (a–c) are significantly different at *p* < 0.05 according to the analysis of variance (ANOVA) and Duncan test.

**Table 9:** Bioaccessible content (µmol/g dry weight) of total and individual glucosinolates in turnip tops (mean ± standard deviation).

Sample	Total	PRO	GNA	GBN	GBS	4OMGBS	Others
Conventional turnip tops (Farm I)	11.2 ± 0.3 ^b^	1.24 ± 0.04 ^c^	9.2 ± 0.2 ^b^	0.51 ± 0.01 ^a^	0.15 ± 0.01 ^a^	0.12 ± 0.02 ^a^	0.04 ± 0.02 ^a^
Conventional turnip tops (Farm II)	13.3 ± 0.5 ^c^	0.81 ± 0.01 ^b^	10.8 ± 0.5 ^c^	1.26 ± 0.04 ^b^	0.15 ± 0.02 ^a^	0.19 ± 0.03 ^a^	0.06 ± 0.02 ^a^
Organic top greens (Farm II)	9.3 ± 0.1 ^a^	0.18 ± 0.02 ^a^	7.4 ± 0.1 ^a^	1.25 ± 0.06 ^b^	0.17 ± 0.00 ^a^	0.18 ± 0.06 ^a^	0.05 ± 0.01 ^a^

PRO (Progoitrin), GNA (Gluconapin), GBN (Glucobrassicanapin), GBS (Glucobrassicin), 4OMGBS (4 Methoxyglucobrassicin). Within each column means with different lowercase letters (a–c) are significantly different at *p* < 0.05 according to the analysis of variance (ANOVA) and Duncan test.

## Data Availability

Not Applicable.

## Supplementary Materials

The following supporting information can be downloaded at: https://www.mdpi.com/article/10.3390/foods11030350/s1, Table S1. Peaks; Figure S1. HPLC chromatograms of glucosinolate profiles in turnip greens and turnip tops of the species Brassica rapa grown under conventional (red) and organic (blue) conditions. Turnip greens: Conventional turnip greens (A) and organic turnip greens (B). Turnip tops: Convent.
