# Supplementary material for: Glucosinolates, Ca, Se Contents, and Bioaccessibility in Brassica rapa Vegetables Obtained by Organic and Conventional Cropping Systems
Source: Foods. 2022 Jan 26;11(3):350. doi: 10.3390/foods11030350 (PMC8834489; doi:10.3390/foods11030350)
Supplement: Supplementary file 1 [file foods-11-00350-s001.zip › foods-1543453 - supplementary.pdf]

Supplementary Materials

Table S1. Peaks.

Number	Name	Retention time
1	Progoitrin	6.2
2	Gluconapin	14.7
3	Glucobrassicanapin	18.8
4	Glucotropaeolin (Internal standard)	19.5
5	Glucobrassicin	21.6
6	4-Methoxyglucobrassicin	23.9

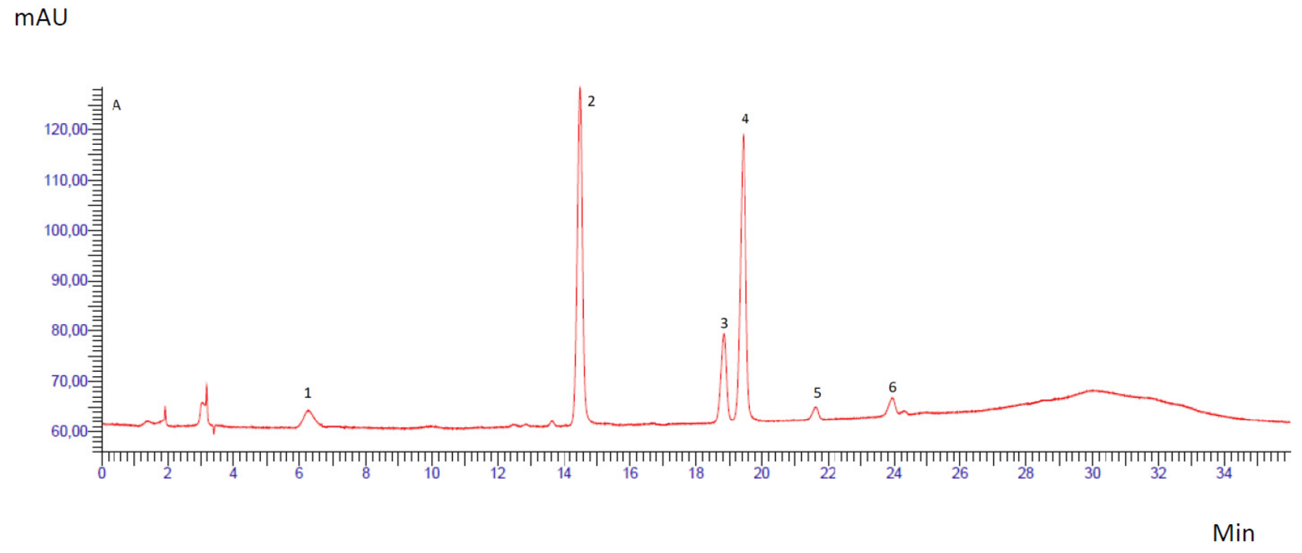

(A)

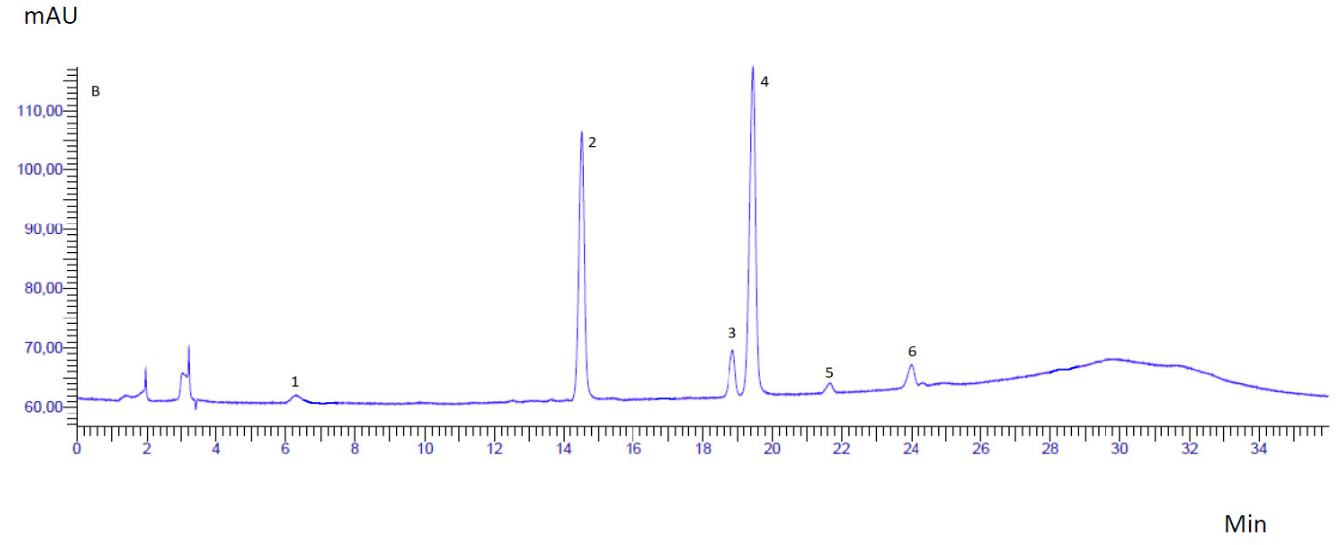

(B)

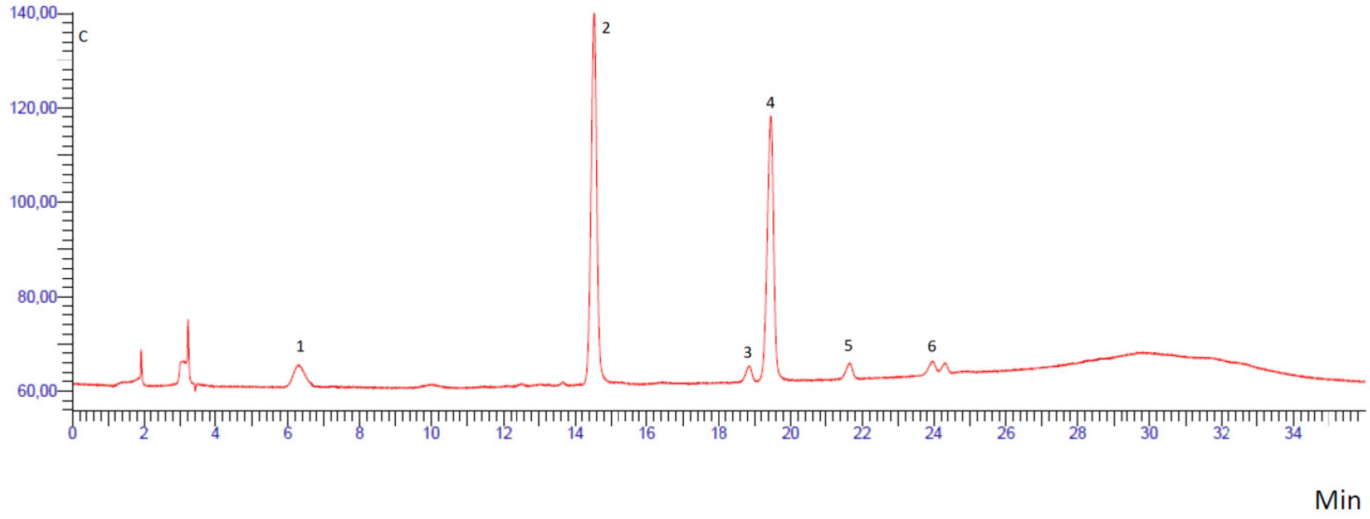

(C)

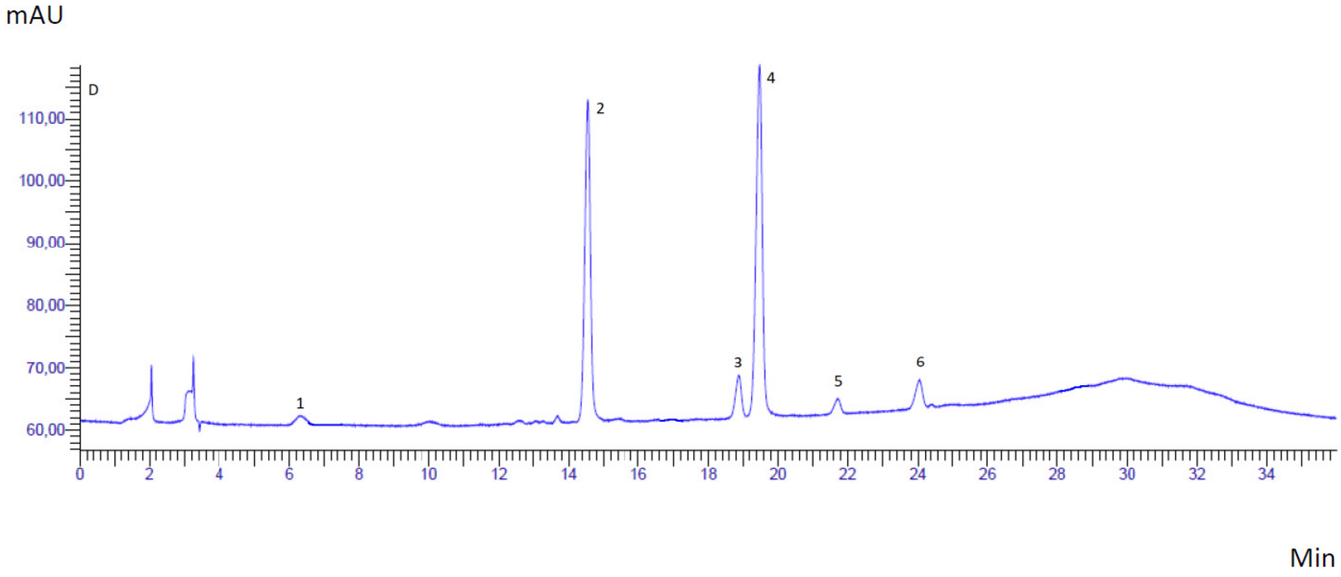

(D)

Figure S1. HPLC chromatograms of glucosinolate profiles in turnip greens and turnip tops of the species *Brassica rapa* grown under conventional (red) and organic (blue) conditions. Turnip greens: Conventional turnip greens (**A**) and organic turnip greens (**B**). Turnip tops: Conventional turnip tops (**C**) and organic turnip tops (**D**).
